# Adult newborn neurons interfere with fear discrimination in a protocol‐dependent manner

**DOI:** 10.1002/brb3.796

**Published:** 2017-08-02

**Authors:** Tzong‐Shiue Yu, Yacine Tensaouti, Zohaib M. Bagha, Rina Davidson, Ahleum Kim, Steven G. Kernie

**Affiliations:** ^1^ Department of Pediatrics Columbia University College of Physicians and Surgeons New York NY USA

**Keywords:** dentate gyrus, fear discrimination, neurogenesis, pattern separation

## Abstract

**Introduction:**

Significant enhancement of neurogenesis is known to occur in response to a variety of brain insults such as traumatic brain injury. Previous studies have demonstrated that injury‐induced newborn neurons are required for hippocampus‐dependent spatial learning and memory tasks like the Morris water maze, but not in contextual fear conditioning that requires both the hippocampus and amygdala. Recently, the dentate gyrus, where adult hippocampal neurogenesis occurs, has been implicated in processing information to form specific memory under specific environmental stimuli in a process known as pattern separation.

**Methods:**

To test whether injury‐induced newborn neurons facilitate pattern separation, hippocampus‐dependent contextual fear discrimination was performed using delta‐HSV‐TK transgenic mice, which can temporally inhibit injury‐induced neurogenesis under the control of ganciclovir.

**Results:**

We observed that impaired neurogenesis enhanced the ability to distinguish aversive from naïve environments. In addition, this occurs most significantly following injury, but only in a context‐dependent manner whereby the sequence of introducing the naïve environment from the aversive one affected the performance differentially.

**Conclusions:**

Temporal impairment of both baseline and injury‐induced adult neurogenesis enhances performance in fear discrimination in a context‐dependent manner.

## 1 INTRODUCTION

Traumatic brain injury (TBI) is a leading cause of death and disability worldwide. Although most people suffering from TBI recover, their quality of life is often poor in part due to hippocampal‐dependent memory‐related disorders (Blennow et al., 2016; Bryant, 2011). Although injury‐induced neurogenesis has been shown to mediate some aspects of recovery following TBI in animal models, mechanisms underlying this remain unclear as do the precise aspects of hippocampal function regulated by injury‐induced neurogenesis.

It is well established that newborn neurons are continually generated throughout life in the hippocampal dentate gyrus and olfactory bulbs (Christian, Song, & Ming, 2014; Ming & Song, 2011). These adult newborn neurons, especially in the dentate gyrus, are believed to participate in particular aspects of spatial and emotional memory formation (Besnard & Sahay, 2016; Christian et al., 2014; Ming & Song, 2011). Several studies also highlight that increases in neurogenesis, like voluntary exercise or exposure to enriched environments, enhance learning and memory formation (Creer, Romberg, Saksida, van Praag, & Bussey, 2010; Garthe, Roeder, & Kempermann, 2016; Thuret, Toni, Aigner, Yeo, & Gage, 2009). Interestingly, enhanced neurogenesis is also observed in a variety of brain injuries and many studies have shown that injury‐induced newborn dentate neurons are essential in recovery from some, though not all, cognitive deficits in animal models of brain injury (Blaiss et al., 2011; Cho et al., 2015; Fernandez‐Hernandez & Rhiner, 2015; Sun, 2016; Sun, Daniels, Rolfe, Waters, & Hamm, 2015).

The hippocampus plays a critical role in encoding similar events (or episodes) discretely and without interfering with previously stored memories (Anacker & Hen, 2017). It has been proposed that a distinct population of neurons in the dentate gyrus are recruited to encode or process information that associate experienced events with corresponding environmental stimuli, known as pattern separation (Amaral, Scharfman, & Lavenex, 2007; Kesner, 2013). Therefore, there is a low level of overlap of neurons in the dentate gyrus that become active even when two sensory stimuli are similar, which ensures the recall of associative memory underlying context‐appropriate responses. Mechanisms for intact pattern separation are required for fundamental aspects of memory formation and integration and many essential cognitive functions go awry if the ability of the dentate gyrus to regulate this is impaired (Besnard & Sahay, 2016; Kheirbek, Klemenhagen, Sahay, & Hen, 2012; Kheirbek, Tannenholz, & Hen, 2012; McHugh et al., 2007). For example, in fear memory, generalization of fear is protective, and an appropriate response to the likelihood of risk in the environment is considered both essential and adaptive. When overgeneralization of fear occurs due to damage in the hippocampus, the ability to discriminate a safe environment from an aversive one is detrimental and can result in posttraumatic stress disorder (PTSD) or panic disorder (Besnard & Sahay, 2016; Kheirbek, Klemenhagen, et al., 2012).

Recent studies show that the ability of mice in distinguishing an aversive environment from a naïve one can be both diminished or enhanced. Impairments are seen when a specific NMDA receptor in adult dentate newborn neurons is selectively deleted, which inactivates these neurons, and enhancements are seen by deleting a proapoptotic gene specifically in adult newborn dentate gyrus neurons, which increases their number (Kheirbek, Klemenhagen, et al., 2012; Kheirbek, Tannenholz et al., 2012; McHugh et al., 2007; Sahay et al., 2011). Hence, newborn neurons in the adult dentate gyrus are believed to regulate pattern separation to ensure correct memory as the response is recalled in the context of specific sensory stimuli.

We used delta‐HSV‐TK transgenic mice to temporally abolish neurogenesis under the control of ganciclovir, and our previous studies demonstrate that injury‐induced newborn neurons in the dentate gyrus facilitate recovery in some, but not all, hippocampal‐dependent tasks (Blaiss et al., 2011; Yu, Kim, & Kernie, 2015; Yu, Zhang, Liebl, & Kernie, 2008). Here, we tested whether injury‐induced newborn neurons in the dentate gyrus facilitate performance in discriminating between aversive and naïve environments. Surprisingly, we found that performance improved in mice when neurogenesis was inhibited whether injury was performed or not. Furthermore, we also observed that the sequence in introducing the aversive and naïve environment affect the performance when neurogenesis was inhibited. Therefore, impaired neurogenesis improved contextual discrimination learning in a protocol‐dependent manner and this phenomenon is enhanced following injury.

## 2 MATERIALS AND METHODS

### 2.1 Animals

All experimental procedures were conducted under the approval of the Institutional Animal Care and Use Committee at Columbia University, and experimental animals were humanely housed and cared for under the supervision of the Institute of Comparative Medicine. The previously generated nestin‐HSV‐TK transgenic mice (Jackson Laboratory #JAX:029671, RRID: IMSR_JAX:029671) on a C57Bl/6 genetic background were used in each experimental groups and no apparent phenotype was observed in the presence of valganciclovir. All mice used were between 8 and 9 weeks of age when injured or mock injured at which time they were fed with either control or valganciclovir chow. Behavioral experiments were started 4 weeks after surgery in order for the injury‐induced neurons to most likely play a role in observed hippocampal behavior (Blaiss et al., 2011; Yu et al., 2015).

### 2.2 Controlled cortical impact injury

The standard protocol using a controlled cortical impact device (Leica Impact One, Leica Biosystems) was used to generate brain injuries as described previously (Blaiss et al., 2011; Yu et al., 2008, 2015). Eight‐ or 9‐week‐old nestin‐HSV‐TK mice were anesthetized with isoflurane and then placed in a stereotactic frame. A midline incision was made, the soft tissues were reflected, and a 5 × 5 mm craniotomy was made between bregma and lambda and 1 mm lateral to the midline. The injury was generated on the left hemisphere with a 3 mm stainless steel tipped impact device with a deformation of 0.7 mm, constant speed of 4.4 m/s, and duration of 300 ms. After surgery, the scalp was fastened with sutures and the mice were allowed to recover. For sham operations, mice received all the procedures noted above, but the impact was not performed.

### 2.3 Administrations of 5′‐bromo‐deoxyuridine and valganciclovir

**Figure 1:**
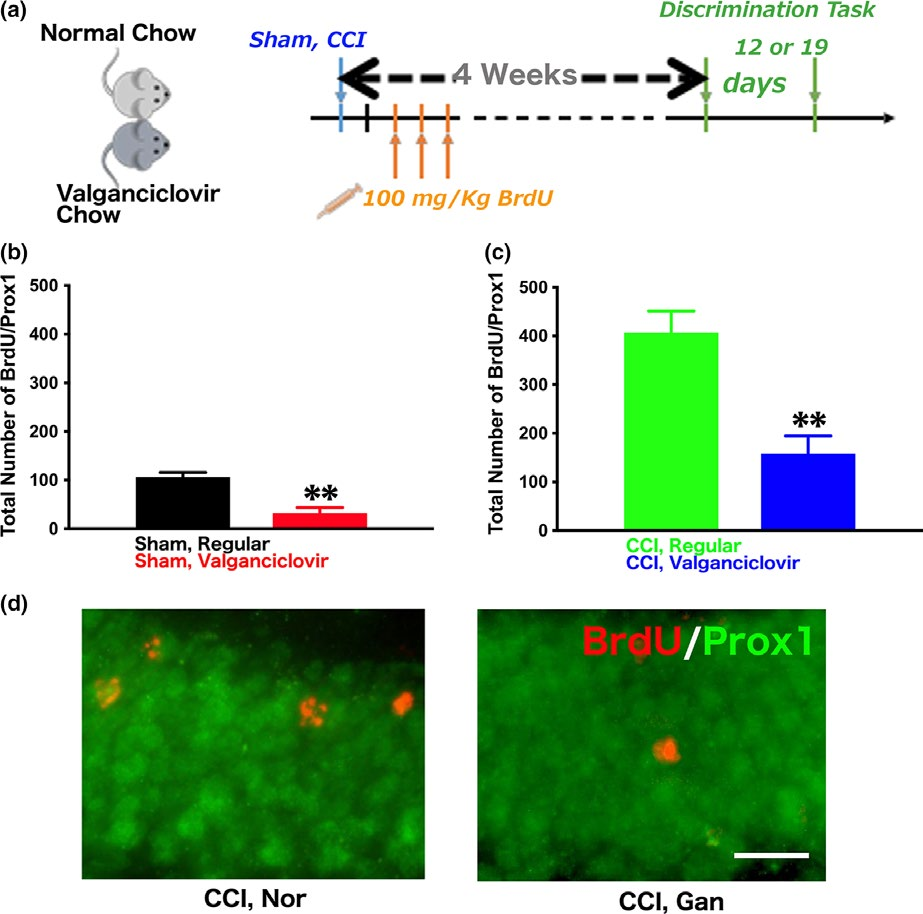
Experimental design and the efficiency of inhibition of neurogenesis. (a) Eight‐week‐old delta‐HSV‐TK transgenic mice were used and underwent sham operation or CCI. One injection of BrdU was given to label the dividing cells for 3 consecutive days 72 hr after surgery. After 4 weeks of recovery, contextual fear discrimination was performed and the task took 13 or 19 days depending on the protocol used. (b) The quantification of newborn neurons in the dentate gyrus represented by BrdU/Prox1‐expressing cells revealed a 75% reduction in sham‐operated mice (unpaired two‐tail *t* test, **p = .0011). (c) The quantification of newborn neurons in the dentate gyrus represented by BrdU/Prox1‐expressing cells revealed a 66% reduction in CCI‐operated mice (unpaired two‐tail *t* test, **p = .001). (d) Representative pictures demonstrate the reduction of injury‐induced newborn neurons after being fed with valganciclovir‐containing chow (scale bar: 20 μm)

5′‐Bromo‐deoxyuridine (BrdU, 100 mg/kg, Sigma) was delivered via intraperitoneal injection once a day for 3 consecutive days starting 72 hr after surgery to verify the efficiency in ablating dividing neuronal progenitors while feeding with valganciclovir‐containing chow. Valganciclovir (Valcyte, Genetech) was mixed with regular grain‐based rodent diet to produce 900 mg/kg valganciclovir‐containing chow (Custom Animal Diets, LLC, Easton, PA) (Yu et al., 2015). Valganciclovir chow was provided ad libitum for valganciclovir‐treated groups until the conclusion of the experiments (Figure 1a). Sham‐operated and injured mice were housed separately to allow approximately equal intake.

### 2.4 Contextual fear‐discrimination learning

The fear discrimination was conducted by an experimenter blinded to the experimental treatment conditions. The protocols of fear discrimination were described by other groups with some modifications (Sahay et al., 2011; Wehner & Radcliffe, 2004). Modifications were primarily in how many times the mice were exposed to A before B was introduced, the sequence of introduction of B to the mice, and total training days.

For fear discrimination learning, all experimental mice were exposed to context A in which a single 2‐s foot shock of 0.75 mA was delivered 3 min after placement in the chamber. Mice were left in the chamber for another 30 s after the shock, then were moved back to their home cage. Depending on experimental groups, mice were exposed to context B either before or after context A with at least a 1‐hr interval. The sequence of exposure in A or B was pseudorandomized and counterbalanced. An automated scoring system (EthoVision XT, Noldus) which digitalized the video signal and compared frame‐by‐frame changes in mouse position was used to measure the freezing levels in both contexts A (3 min before shock) and B (3 min in the chamber) each day.

**Figure 2:**
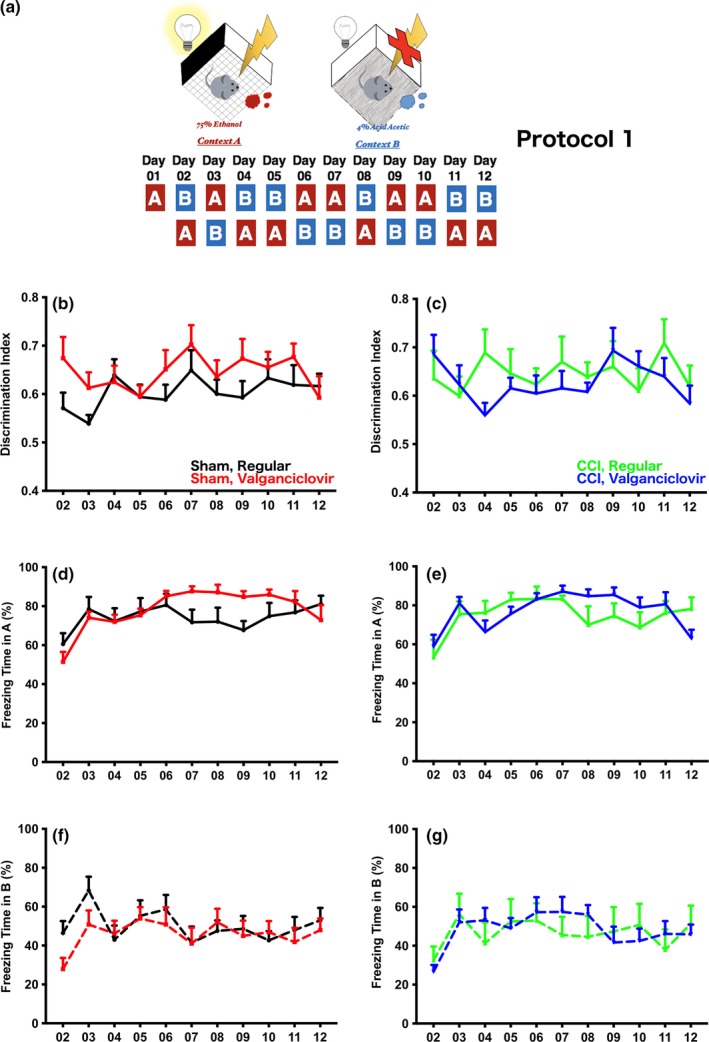
(a) The sequence in exposing mice to contexts A and B in protocol 1. (b) The discrimination index indicated that the learning ability of sham‐operated mice changed along the learning period whether or not neurogenesis was intact, however, there was no significant difference between mice with intact neurogenesis and mice with impaired neurogenesis (*p* = .0297 with learning day as the variable, *p* = .233 with experimental group as the variable, and *p* = .3209 in the interaction in repeated measures two‐way ANOVA with Sidak's multiple comparisons). (c) A similar result was observed in the injured mice, the discrimination index suggested that no significant difference was found in injured mice regardless of whether or not neurogenesis was intact. Different from the sham‐operated mice, however, the index was not changed along the learning period in the injured mice (*p* = .2790 with learning day as the variable, *p* = .6057 with experimental group as the variable, and *p* = .1610 in the interaction in repeated measures two‐way ANOVA with Sidak's multiple comparisons). (d) To further investigate the performance of mice in context A, the percentage of freezing time along the learning period was analyzed. Consistent with the discrimination index, no significant difference was observed in sham‐operated mice with or without intact neurogenesis (*p* < .0001 with learning day as the variable, *p* = .4295 with experimental group as the variable, and *p* < .0015 in the interaction in repeated measures two‐way ANOVA with Sidak's multiple comparisons). (e) Similar to sham‐operated mice, the performance of injured mice was alike in context A regardless of whether or not neurogenesis was intact (*p* < .0001 with learning day as the variable, *p* = .6639 with experimental group as the variable, and *p* = .0424 in the interaction in repeated measures two‐way ANOVA with Sidak's multiple comparisons). (f) In context B, sham‐operated mice with impaired neurogenesis performed similarly as sham‐operated mice with intact neurogenesis (*p* < .0001 with learning day as the variable, *p* = .5722 with experimental group as the variable, and *p* = .1366 in the interaction in repeated measures two‐way ANOVA with Sidak's multiple comparisons). (g) A similar performance was observed in injured mice whether or not neurogenesis was intact (*p* = .0003 with learning day as the variable, *p* = .8902 with experimental group as the variable, and *p* = .2903 in the interaction in repeated measures two‐way ANOVA with Sidak's multiple comparisons)

**Figure 3:**
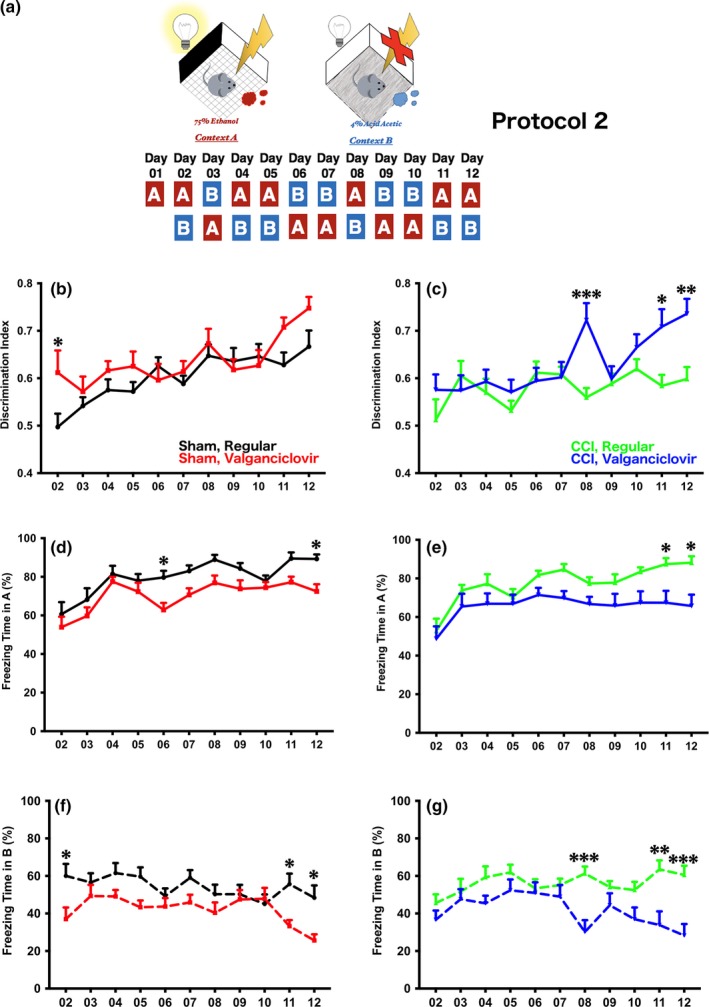
(a) The arrangement of contexts A and B, and the sequence of exposure to both contexts in protocol 2 (opposite to protocol 1). (b) Different from what was observed when protocol 1 was used, a subtle difference was observed in the sham‐operated mice with impaired neurogenesis. The mice showed discrimination at day 2, the first time context B was introduced (*p* < .0001 with learning day as the variable, *p* = .1233 with experimental group as the variable, and *p* = .0610 in the interaction in repeated measures two‐way ANOVA; *p < .05 in Sidak's post hoc analysis). (c) In injured mice, the discrimination index revealed that injured mice without neurogenesis had enhanced discrimination (*p* < .0001 with learning day as the variable, *p* = .1233 with experimental group as the variable, and *p* = .0610 in the interaction in repeated measures two‐way ANOVA; *p < .05, **p < .01, ***p < .001 in Sidak's post hoc analysis). (d and e) The freezing time in context A demonstrated that mice without intact neurogenesis spent significantly less freezing time whether injured or not (d for sham‐operated mice, *p* < .0001 with learning day as the variable, *p* = .001 with experimental group as the variable, and *p* = .5647 in the interaction in repeated measures two‐way ANOVA; *p < .05 in Sidak's post hoc analysis; e for injured mice, *p* < .0001 with learning day as the variable, *p* = .0114 with experimental group as the variable, and *p* = .2650 in the interaction in repeated measures two‐way ANOVA; *p < .05 in Sidak's post hoc analysis). (f and g) A similar decrease in freezing time in both sham‐operated and injured mice without intact neurogenesis in context B (f for sham‐operated mice, *p* = .0022 with learning day as the variable, *p* = .0069 with experimental group as the variable, and *p* = .0287 in the interaction in repeated measures two‐way ANOVA; *p < .05 in Sidak's post hoc analysis; g for injured mice, *p* = .0034 with learning day as the variable, *p* = .0128 with experimental group as the variable, and *p* < .0001 in the interaction in repeated measures two‐way ANOVA; **p < .01, ***p < .001 in Sidak's post hoc analysis)

**Figure 4:**
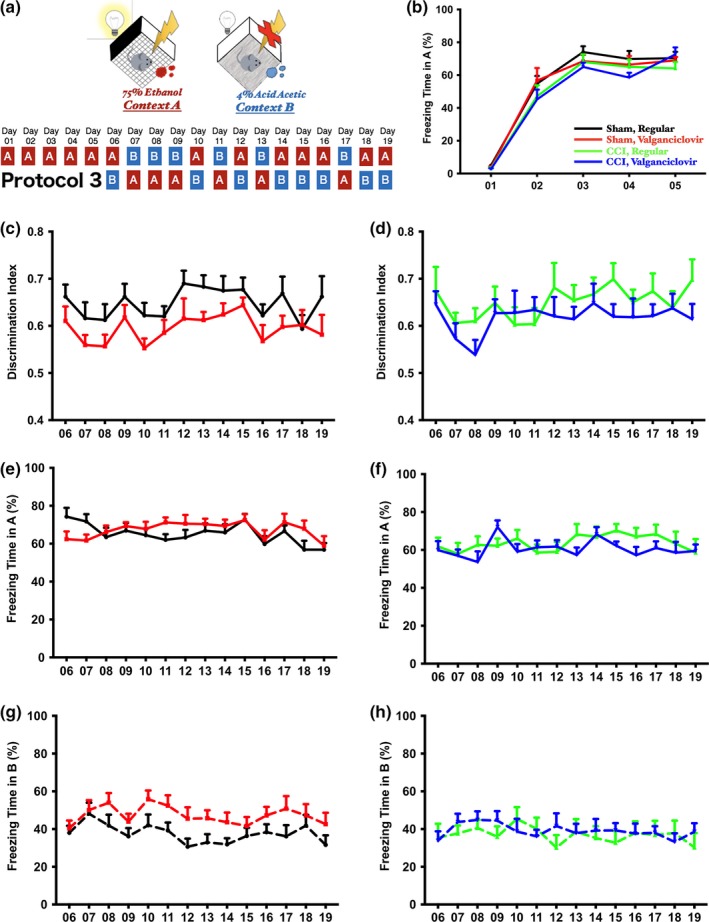
(a) The arrangement of contexts A and B, and the sequences of exposure to both contexts in protocol 3 was similar to protocol 2. (b) Mice associated the foot shock with context A successfully in a 5‐day training session regardless of injury or whether neurogenesis was intact or not (repeated measures two‐way ANOVA with Tukey's multiple comparisons; time effect, *p* < .0001; experimental groups, *p* = .2306). (c and d) After the 5‐day training, there was no significant difference in indices using protocol 2 regardless of injury status or whether or not neurogenesis was intact (c for sham‐operated mice, *p* = .0094 with learning day as the variable, *p* = .0207 f with experimental group as the variable, *p* = .9760 for the interaction using repeated measures two‐way ANOVA; d for injured mice, *p* = .0773 with learning day as the variable, *p* = .2725 with experimental group as the variable, *p* = .7326 for the interaction using repeated measures two‐way ANOVA). (e and f) After extensive training, the decrease in freezing time in context A in both sham‐operated and injured mice with impaired neurogenesis when protocol 2 was used was not observed (e for sham‐operated mice, *p* = .0299 with learning day as the variable, *p* = .2630 with experimental group as the variable, *p* = .1726 for the interaction using repeated measures two‐way ANOVA; f for injured mice, *p* = .1221 with learning day as the variable, *p* = .4552 with experimental group as the variable, *p* = .2159 for the interaction using repeated measures two‐way ANOVA). (g and h) After extensive training, the decrease in freezing time in context B in both sham‐operated and injured mice with impaired neurogenesis when protocol 2 was used was not observed (g for sham‐operated mice, *p* = .0319 with learning day as the variable, *p* = .0176 with experimental group as the variable, *p* = .9205 for the interaction using repeated measures two‐way ANOVA; h for injured mice, *p* = .6211 with learning day as the variable, *p* = .6931 with experimental group as the variable, *p* = .5198 for the interaction using repeated measures two‐way ANOVA)

Three different protocols were used to verify the impact of newborn neurons in discrimination (Figures 2a, 3a, 4a). In the extensive learning protocol (Figure 4a, protocol 3), experimental mice were exposed to context A with a 2‐s foot shock every day for further habituation, and context B was introduced starting from day 6 (the learning session). In the brief exposure version, two protocols were used. In both protocols, mice were exposed to context A with a 2‐s foot shock without exposure to B in the first day (conditioning). Starting on day 2, context B was introduced either before (Figure 2a, protocol 1) or after (Figure 3a, protocol 2) context A with at least a 1‐hr interval separating them. The sequence of exposure to A and B in protocol 2 were opposite to protocol 1.

### 2.5 Immunohistochemistry

All mice were deeply anesthetized with isoflurane through the whole period of perfusion. The transcardiac perfusion was performed with 50 ml of 1x PBS, followed by 100 ml of 4% paraformaldehyde (PFA) in 1x PBS. Whole brains were then dissected and immersed in 4% PFA/1x PBS overnight for postfixation. Then, brains were embedded in 3% agarose/1x PBS and serial 50 μm sections were cut with a vibratome (VT1000S, Leica). All sections encompassing the hippocampus were collected sequentially in six‐well plates. Free‐floating sections were used for immunohistochemistry. For BrdU staining, all sections from a single well were washed with 1x PBS three times and rinsed with water. Then, sections were denatured with 1 N HCl for 45 min in 37°C water bath. After denaturation, sections were washed with 0.3% Triton X‐100/1x PBS for three times, 10 min each time. Then, sections were blocked with 5% normal donkey serum (017‐000‐121, Jackson ImmunoResearch Laboratories) in 0.3% Triton X‐100/1x PBS for 1 hr at room temperature. Rat anti‐BrdU antibody (1:500; Abcam Cat# ab6326 RRID:AB_305426) and rabbit anti‐prox1 antibody (1:500; Abcam Cat# ab101851 RRID:AB_10712211) were used to label BrdU and mature dentate neurons overnight at 4°C. The following day, sections were incubated with Alexa‐488‐conjugated donkey anti‐rabbit and Alexa‐594‐conjugated donkey anti‐rat antibodies (both 1:200, Jackson ImmunoResearch) for 3 hr at room temperature to visualize Prox1 and BrdU, respectively. The sections were placed on the slides and covered with coverslips.

### 2.6 Quantification and microscopy

To quantify BrdU and BrdU/Prox1 cells, we used a traditional and well‐established method to account for oversampling as described before with modifications (Miles & Kernie, 2008; Yu et al., 2015). A Zeiss Imager M2 microscopy using Zeiss Neofluor 40x/1.3 oil lens was used to determine BrdU‐labeled cells and colocalization of BrdU and Prox1. Cell quantification was performed from the ipsilateral and contralateral sides of the dentate gyrus in injured and sham‐operated mice. We sampled a well of sections 50‐μm thick, coronally sectioned, and PFA‐fixed 300 μm apart. Sections from each well were stained with BrdU and Prox1 as free‐floating sections to allow equal antibody penetration between sections. Sections were mounted using Immunomount (Vectashield D, Vector Labs) with a refractive index of 1.495. Antibody penetration and signal intensity were found to be evenly distributed throughout the *z*‐axis in multiple sections from different mice. Each blade of the dentate gyrus was counted separately at 40x under Zeiss immersion oil 518F. Blinded quantification was performed on BrdU alone and BrdU/Prox1 fully colocalized cells in the granular layer of the dentate gyrus. By scanning serially through the *z*‐axis of each cell, a more precise discrimination of the colocalization of relevant cell markers was permissible. Since 1/6 of the total number of sections was counted, we multiplied our counts by 6 to obtain the number of cells per dentate gyrus.

### 2.7 Statistics

An unpaired two‐tailed *t* test was used to determine the significant decrease in the number of newborn neurons after valganciclovir chow was given. Whether mice were able to distinguish two similar contexts was determined by the discrimination index. The discrimination index was calculated with the following formula: (freezing time in A)/(freezing time in A + freezing time in B). Analyses of the discrimination index and freezing time in context A or B were done using repeated measures two‐way ANOVA with Sidak's multiple comparisons. All the data were presented as mean ± *SEM*.

## 3 RESULTS

### 3.1 Valganciclovir inhibits neurogenesis in nestin‐HSV‐TK mice

We inducibly inhibited neurogenesis with previously generated and well‐characterized delta‐HSV‐TK transgenic mice (Blaiss et al., 2011; Yu et al., 2008, 2015). Eight‐week old mice underwent either a sham operation or CCI (Figure 1a). Three days after surgery, BrdU was injected once per day for 3 consecutive days to label proliferating neural progenitors (Blaiss et al., 2011; Yu et al., 2008, 2015). Starting from the day surgery was performed, mice were randomly assigned to be fed with either regular chow or chow mixed with valganciclovir until the experiment was concluded (Figure 1a). The discrimination learning task started 4 weeks after surgery, and three groups of mice were used for three different learning protocols. To ensure the efficiency of valganciclovir administration for inhibiting neurogenesis, immunofluorescent staining was performed to label BrdU and Prox1, a dentate gyrus‐specific neuronal marker, in brain sections collected from all mice fed with valganciclovir chow and samples were randomly chosen from each experimental group for quantification of newborn neurons represented by BrdU/Prox1‐labeled cells. Treatment with valganciclovir resulted in a significant inhibition of neurogenesis regardless of whether the mice underwent a sham or CCI procedure (Figure 1b–d). In sham‐operated mice, the reduction in the number of newborn neurons was roughly 75% on both sides of the dentate gyrus (Figure 1b, two‐tailed unpaired *t* test, *p* = .0011). In injured groups, the decrease was approximately 66% (Figure 1c and d, two‐tailed unpaired *t* test, *p* = .0011). Therefore, we concluded that HSV‐TK mice treated with valganciclovir caused a significant reduction in the number of newborn neurons in the dentate gyrus in both sham‐operated and injured mice.

### 3.2 Intact neurogenesis is not required for fear discrimination when the aversive stimulus is presented first

To investigate whether adult newborn neurons play a role in discriminating an aversive environment from a naïve one that shares similar cues, three protocols were used in contextual fear discrimination tasks. We first introduced context A to mice with an aversive foot shock at day 1 without exposure to the naïve context B (Figure 2a). Starting from day 2, context B was introduced to all experimental mice and context B was introduced before A in protocol 1 (Figure 2a). No significant difference was observed in sham‐operated mice without intact neurogenesis when the discrimination index was used as an indicator for distinguishing aversive from naïve contexts (Figure 2b, *p* = .0297 with learning day as the variable, *p* = *.233* with experimental group as the variable, and *p* = .3209 in the interaction in repeated measures two‐way ANOVA with Sidak's multiple comparisons). The same phenomenon was also observed in injured mice even when neurogenesis was impaired (Figure 2c, *p* = .2790 with learning day as the variable, *p* = .6057 with experimental group as the variable, and *p* = .1610 in the interaction in repeated measures two‐way ANOVA with Sidak's multiple comparisons). When the freezing of mice in context A revealed by percentage of freezing time was examined in sham‐operated mice, once again no significant difference was observed in mice with or without intact neurogenesis (Figure 2d, *p* < .0001 with learning day as the variable, *p* = .4295 with experimental group as the variable, and *p* < .0015 in the interaction in repeated measures two‐way ANOVA with Sidak's multiple comparisons). The same result was observed in injured mice (Figure 2e, *p* < .0001 with learning day as the variable, *p* = .6639 with experimental group as the variable, and *p* = .0424 in the interaction in repeated measures two‐way ANOVA with Sidak's multiple comparisons). As expected, all experimental mice demonstrated similar freezing in context B regardless of treatment (Figure 2f for sham‐operated mice, *p* < .0001 with learning day as the variable, *p* = .5722 with experimental group as the variable, and *p* = .1366 in the interaction; Figure 2g for injured mice, *p* = .0003 with learning day as the variable, *p* = .8902 with experimental group as the variable, and *p* = .2903 in the interaction in repeated measures two‐way ANOVA). Hence, all experimental mice demonstrated similar freezing times in contexts A and B in distinguishing the naïve context B from the aversive context A when protocol 1 was used.

### 3.3 Fear discrimination is impaired when neurogenesis is inhibited when the naïve stimulus is encountered first

In protocol 2, we reversed the sequence of A and B when compared to protocol 1 (Figure 3a), and found that the freezing times were quite different. In sham‐operated mice, those without intact neurogenesis showed a significant improvement in discrimination the first day when context B was introduced (Figure 3b, *p* < .0001 with learning day as the variable, *p* = .1233 with experimental group as the variable, and *p* = .0610 in the interaction in repeated measures two‐way ANOVA; *p < .05 in Sidak's post hoc analysis). However, mice with and without neurogenesis showed similar abilities in discrimination after day 2. In injured mice, an apparent improvement in discrimination was observed in mice with impaired neurogenesis when compared with injured mice with intact neurogenesis (Figure 3c, *p* < .0001 with learning day as the variable, *p* = .1233 with experimental group as the variable, and *p* = .0610 in the interaction in repeated measures two‐way ANOVA; *p < .05, **p < .01, ***p < .001 in Sidak's post hoc analysis). To understand why an improved discrimination index was observed in mice with impaired neurogenesis, the freezing times in contexts A and B were examined. In sham‐operated mice, mice without intact neurogenesis spent less freezing time in both contexts A and B (Figure 3d and f, d for context A, *p* < .0001 with learning day as the variable, *p* = .001 with experimental group as the variable, and *p* = .5647 in the interaction in repeated measures two‐way ANOVA; ^*^ *p* < .05 in Sidak's post hoc analysis; f for context B, *p* = .0022 with learning day as the variable,, *p* = .0069 with experimental group as the variable, and *p* = .0287 in the interaction in repeated measures two‐way ANOVA; ^*^ *p* < .05 in Sidak's post hoc analysis). A similar but more apparent outcome was observed in the injured groups (Figure 3e and f). In context A, injured mice with impaired neurogenesis demonstrated less freezing time (Figure 3e, *p* < .0001 with learning day as the variable, *p* = .0114 with experimental group as the variable, and *p* = .2650 in the interaction in repeated measures two‐way ANOVA; *p < .05 in Sidak's post hoc analysis). A similar pattern of freezing time was observed in context B (Figure 3g, *p* = .0034 with learning day as the variable, *p* = .0128 with experimental group as the variable, and *p* < .0001 in the interaction in repeated measures two‐way ANOVA; **p < .01, ***p < .001 in Sidak's post hoc analysis). Hence, less neurogenesis resulted in a weaker association of aversive stimulus with context A and more activity in the naïve context B when protocol 2 was used.

### 3.4 Overtraining in the aversive context A eliminates the effect of impairing neurogenesis

To rule out the possibility that mice might not associate an aversive foot shock with context A when protocol 2 was used, we exposed mice in context A with an aversive foot shock for 5 consecutive days before context B was introduced. The naïve context B was introduced after exposure of context A on day 6, but the exposure sequence was similar to protocol 2 (Figure 4a). After a 5‐day training session, mice associated the aversive foot shock with context A successfully as indicated by similar percentage of freezing time in context A (Figure 4b, repeated measures two‐way ANOVA with Tukey's multiple comparisons; time effect, *p* < .0001; experimental groups, *p* = .2306). As expected, the discrimination index demonstrated that sham mice were able to distinguish context B from A whether neurogenesis was intact or not (Figure 4c, *p* = .0094 with learning day as the variable, *p* = .0207 with experimental group as the variable, *p* = .9760 for the interaction using repeated measures two‐way ANOVA). The ability in distinguishing contexts A and B in injured mice with or without intact neurogenesis was similar (Figure 4d, *p* = .0773 with learning day as the variable, *p* = .2725 with experimental group as the variable, *p* = .7326 for the interaction using repeated measures two‐way ANOVA). After examining the freezing time in both contexts, sham‐operated mice with or without intact neurogenesis spent similar freezing times in contexts A and B (Figure 4e and g). Moreover, similar freezing times were observed in injured mice whether they retained intact neurogenesis or not (Figure 4f and h). Therefore, the enforcement in association between aversive stimulus and corresponding context A diminished the phenomenon observed in mice with impaired neurogenesis when protocol 2 was used. Hence, we conclude that adult newborn neurons interfere with contextual discrimination in a protocol‐dependent manner.

## 4 DISCUSSION

Patients who recover from traumatic brain injury often suffer a variety of cognitive deficits, and treatments for these deficits remain supportive (Blennow et al., 2016; Hemphill, Dauth, Yu, Dabiri, & Parker, 2015; Yu, Washington, & Kernie, 2016). The recognition that injury‐induced neurogenesis occurs and appears to mediate some aspects of cognitive recovery, especially in the dentate gyrus in the hippocampus which is involved in learning and memory, raises the hope that manipulating neurogenesis might improve learning and memory after brain injury (Akers et al., 2014; Arruda‐Carvalho, Sakaguchi, Akers, Josselyn, & Frankland, 2011; Deng, Aimone, & Gage, 2010; Johnston, Shtrahman, Parylak, Goncalves, & Gage, 2016; Massa et al., 2011; Stone et al., 2011). Using mouse models to mimic traumatic brain injury, we previously demonstrated that injury‐induced neurogenesis is essential in spatial learning and memory after brain injury, but it does not appear to be required in contextual fear conditioning (Blaiss et al., 2011). It has been proposed that contextual fear conditioning is not a hippocampus‐dependent task and that the amygdala plays a critical role in mediating this particular kind of memory (Wehner & Radcliffe, 2004). Therefore, we employed the discrimination version of the contextual fear task, which is highly hippocampal dependent (Frankland, Cestari, Filipkowski, McDonald, & Silva, 1998; Wehner & Radcliffe, 2004) in order to determine the requirement of injury‐induced newborn neurons in successfully differentiating aversive and naïve contexts after TBI.

We observed that using a protocol where context B was introduced before the aversive context A at day 2 (protocol 1), mice were able to differentiate aversive and naïve contexts regardless of treatment. By using a different protocol where context A was introduced before B at day 2 (protocol 2), mice lacking neurogenesis unexpectedly retained higher discrimination indices regardless of whether they were injured or not. Moreover, both sham‐operated and injured mice lacking neurogenesis spent significantly less freezing time in both contexts A and B, though the observation was enhanced in the setting of CCI. This may be due to the increase in neurogenesis that occurs following injury compared with sham‐injured mice (Figure 1), which supports the observation that more relative impairment of neurogenesis results in improved fear discrimination as demonstrated by a higher discrimination index in mice who are both injured and have impaired neurogenesis (Figure 3c). Extensive exposure to mice to the aversive foot shock in context A for 5 consecutive days (protocol 3) eliminated the impairment of fear discrimination memory observed with ablating neurogenesis seen in protocol 2 (Figure 4).

Decreases in adult hippocampal neurogenesis impair pattern separation, whereas increases facilitate it (Clelland et al., 2009; Clemenson et al., 2015; Sahay et al., 2011; Tronel et al., 2012). However, our observations using various fear discrimination paradigms to test pattern separation are not consistent with this model. When we introduced the naïve context B before A in protocol 1, no significant difference was observed among experimental groups in the discrimination index. Based on data using this protocol alone, one may conclude that adult newborn neurons are not required in learning contextual discrimination. However, sham‐operated and injured mice with impaired neurogenesis spent less freezing time in both contexts A and B compared with mice with intact neurogenesis when context B was introduced after A at day 2. The reduction of freezing time in both contexts A and B in mice with impaired neurogenesis could result in an increase in the discrimination index, which suggests an improvement in contextual discrimination. Therefore, using the discrimination index alone to determine whether mice learn to distinguish aversive and naïve contexts might be misleading without further examining freezing time in the individual contexts of A and B. Furthermore, the performance of experimental mice varied according to the specific protocol employed. To our knowledge, this is the first report suggesting that variance of performance in contextual fear discrimination might be protocol dependent. Several studies raise similar concerns for contextual fear conditioning, although this task includes both hippocampal and amygdala‐dependent functions and its regulation appears more complicated than that for fear discrimination (Fujinaka et al., 2016; Huckleberry, Ferguson, & Drew, 2016). Hence, further investigation is needed to propose a standard protocol for contextual fear discrimination.

By performing extensive training, mice without intact neurogenesis associated the aversive stimulus with the corresponding context A successfully and were able to distinguish the naïve context B from the aversive context A during learning independent of injury. Interestingly, a previous study demonstrated that adult‐born neurons are necessary for extended contextual discrimination by employing a doxycycline‐dependent means of inducing overexpression of the proapoptotic protein, Bax, to trigger apoptosis in neural progenitors and thus inhibit neurogenesis (Tronel et al., 2012). Although they reported a lesser discrimination ratio in mice with impaired neurogenesis, details using a post hoc analysis were not reported and therefore, it is not clear if a significant difference existed between experimental groups along the 8‐day learning period. It is also possible that the two extra training days given in our study resulted in a more vivid association of the aversive stimulus with context A, which allowed mice to distinguish context B from A more easily whether or not their brains were injured or neurogenesis was intact.

Without intact neurogenesis, mice spent less freezing time in both contexts A and B when protocol 2 was used. The observed deficit was reversed when mice were trained to associate the aversive foot shock with context A successfully before introduction of the naïve context B. Therefore, the observed deficit in protocol 2 is due to a weak association of the foot shock with context A. However, this cannot explain what was observed when protocol 1 was used. Interestingly, the performance of mice, even controls, in contexts A and B varied in several publications according to various published protocols (Clemenson et al., 2015; Sahay et al., 2011; Tronel et al., 2012; Wu & Hen, 2014). The present study highlights the difficulty encountered in ascribing generalized phenomena such as fear discrimination in mice to particular aspects of either baseline or enhanced (by injury) adult hippocampal neurogenesis when the behavioral paradigms used are so exquisitely protocol dependent.

## CONFLICT OF INTEREST

The authors declare no competing financial interests.
